# A bibliometric analysis of Kallmann syndrome: trends, hotspots, and future directions

**DOI:** 10.1186/s13023-026-04378-3

**Published:** 2026-05-18

**Authors:** Hailei Han, Bin Ma, Jinmi Liu, Donglin Chai, Qinghua Sun, Ruiqing Zhang, Yanqing Guo

**Affiliations:** 1 Center for Geriatric Medicine, Shanxi Provincial Geriatric Hospital, Taiyuan, Shanxi China; 2 First Hospital of Shanxi Medical University, Taiyuan, Shanxi China

**Keywords:** Kallmann syndrome, Bibliometrics, Visualization, CiteSpace, VOSviewer

## Abstract

**Background:**

Kallmann syndrome (KS) is a rare congenital disorder characterized by hypogonadotropic hypogonadism secondary to deficient gonadotropin-releasing hormone (GnRH) secretion, often accompanied by partial or complete anosmia. Deficient GnRH secretion results in decreased levels of follicle-stimulating hormone (FSH) and luteinizing hormone (LH), causing impaired sexual development and absent secondary sexual characteristics. Against the backdrop of global expansion in rare disease research, KS has become a core research focus, and a comprehensive bibliometric analysis is required to chart the trajectory, trends, hotspots, and future paths in KS research over the last 15 years.

**Methods:**

A systematic search of the Web of Science Core Collection (WOSCC) retrieved KS publications from January 1, 2009, to August 18, 2025, employing the search term “Kallmann syndrome” with the language restricted to English and no restrictions imposed on publication types. Data analyses were performed using VOSviewer for visualizing collaborative networks, CiteSpace for bursts and clusters, and R for statistics, to evaluate publication trends, countries, institutions, authors, journals, citations, and keywords.

**Results:**

KS publications exhibited steady double-digit annual growth, reaching 508 studies. The U.S. ranked first in publication volume, followed by Switzerland and France. Nelly Pitteloud (Switzerland) and Jacques Young (Paris Public Hospitals) were the most prolific and influential authors. The Journal of Clinical Endocrinology and Metabolism published the largest number of KS-related articles; Harvard University was the leading contributing institution. The most highly cited article, “Expert Consensus: European Consensus Statement on Congenital Hypogonadotropic Hypogonadism—Pathogenesis, Diagnosis and Treatment,” was cited 575 times in total. Keywords identified hotspots like GnRH deficiency, hypogonadotropic hypogonadism, anosmia, and genetic mutations (> 30 genes), current research frontiers focusing on molecular pathogenesis and personalized therapies.

**Conclusions:**

Our study provided a comprehensive overview of Kallmann syndrome research and showed the development status and scientific trend of Kallmann syndrome through bibliometric analysis from 2009 to 2025. The global volume of publications related to Kallmann syndrome has demonstrated a steady year-on-year increase. Research in this field is predominantly led by European and American countries, and cross-regional collaboration serves as a key driver for further advancement. In summary, these findings provide new perspectives for future relevant research and serve as a valuable reference to guide researchers in subsequent studies.

**Supplementary Information:**

The online version contains supplementary material available at 10.1186/s13023-026-04378-3.

## Introduction

Kallmann syndrome (KS) is a genetically heterogeneous disorder characterized primarily by the co-occurrence of hypogonadotropic hypogonadism and anosmia or hyposmia, which is frequently associated with inadequate gonadotropin-releasing hormone (GnRH) secretion and olfactory bulb hypoplasia [1, 2]. Similar to other forms of congenital hypogonadotropic hypogonadism, KS is defined by impaired or delayed pubertal development, leading to clinical manifestations such as microorchidism, primary amenorrhea (with failure to achieve menarche in females), sparse pubic hair, and inadequate breast development. Affected individuals may also present at birth with micropenis or cryptorchidism. These features arise from insufficient secretion of luteinizing hormone (LH) and follicle-stimulating hormone (FSH), resulting in reduced testosterone levels in males and diminished estrogen and progesterone levels in females [3]. Additional phenotypes can include cleft lip or palate, unilateral renal agenesis, cryptorchidism, micropenis, and neurological anomalies such as central auditory impairment, mirror movements (synkinesis), and cerebellar ataxia [4, 5].

KS is typically inherited in an X-linked recessive pattern, with an incidence approximately 4–5 times higher in males than in females. This rare condition, often diagnosed during adolescence due to delayed puberty, has an estimated prevalence of 1 in 48,000 individuals overall (1 in 30,000 males and 1 in 125,000 females) [6, 7]. Therapeutic goals focus on inducing secondary sexual characteristics, maintaining bone mineral density, preserving muscle mass, and restoring fertility. Management commonly involves hormone replacement therapy, such as testosterone in males or estrogen-progesterone in females, with lifelong androgen supplementation often required [4]. Untreated or delayed puberty can lead to significant emotional, psychological, psychosexual, and social consequences [8, 9]. Spontaneous reversal of hypogonadotropic hypogonadism occurs in 10–20% of cases, potentially restoring normal hormonal function, though this recovery may not be permanent [10–12]. The mechanisms underlying this intermittent reversal remain elusive, highlighting the need for further mechanistic studies to improve prognosis in this rare disorder.

Bibliometric analysis is a qualitative and quantitative methodology that applies statistical and mathematical techniques to analyze publications in a specific field over a defined period, producing visual and tabular representations [13]. It provides insights into annual publication volumes, citation frequencies, article types, journal categories, author and national contributions, and keyword trends to identify research hotspots and future directions [14]. This method is especially valuable for summarizing research progress in rare endocrine disorders, where small patient cohorts limit large-scale clinical studies. The present study examines the developmental trajectory of KS research over the past 15 years, focusing on recent advancements. Notably, no comprehensive bibliometric analysis focusing specifically on KS has been published to date. Therefore, this study employs CiteSpace, VOSviewer, and R tools in RStudio to analyze KS-related publications, elucidating trends and focal areas. Through this bibliometric approach, we aim to identify leading authors, institutions, and keywords, offering valuable references for advancing the understanding of KS mechanisms, diagnosis, and treatment.

## Materials and methods

### Database selection

This study utilized the Web of Science Core Collection (WOSCC) database for data analysis. WOSCC was chosen for its widespread academic application, high data authority and standardized structure, which are optimal for biomedical bibliometric analyses.

### Search strategy

The search strategy employed in this study utilized the following keywords: “Kallmann syndrome” with language restrictions set to English. The retrieval timeframe was established from January 1, 2009 to August 18, 2025. Document types were not restricted, to ensure a comprehensive and objective overview of the field.

**Fig. 1:**
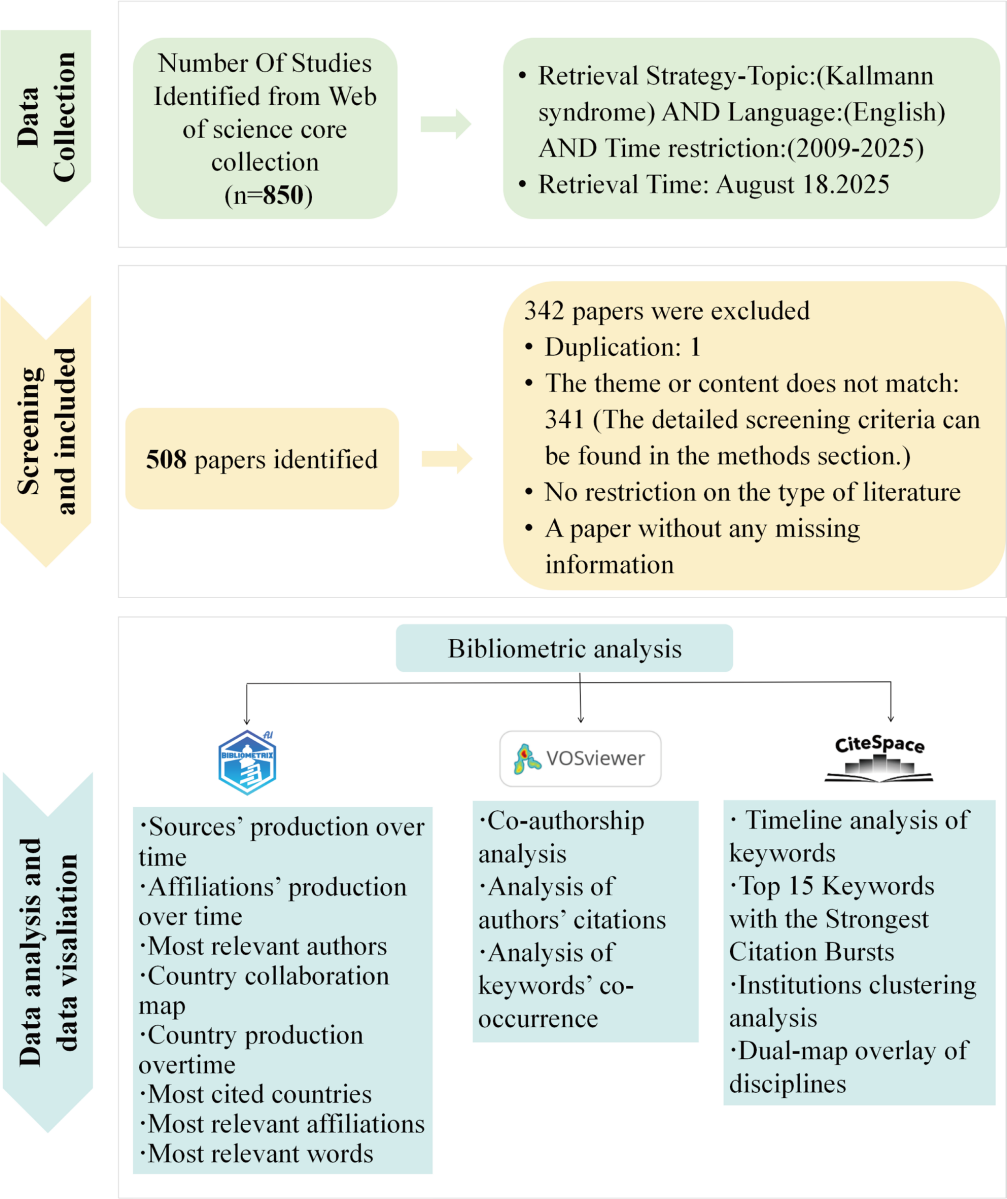
Flowchart of the bibliometric analysis conducted in this study, including search databases, search strategies, study inclusion criteria, and software applications

The Web of Science Core Collection included 850 English-language publications published from 2009 to 2025. After manual screening, a total of 342 publications were excluded. Among these, 1 duplicate record was removed, and 341 publications were excluded due to irrelevant themes or content. The specific inclusion and exclusion criteria are detailed below. The inclusion criterion was papers focused on Kallmann syndrome. The exclusion criteria were as follows: (i) papers on diseases with reduced gonadal function (Congenital hypogonadotropic hypogonadism (CHH)), but not related to Kallmann syndrome (e.g., Klinefelter syndrome, CHARGE syndrome, etc.); (ii) papers with missing information (e.g., author details, journal name, or publication year). We did not set restrictions on the research design (e.g., observational, experimental) or publication type (e.g., editorial, original article, review). According to the inclusion and exclusion criteria, a total of 508 papers were included. As this study falls within the scope of bibliometrics, no ethical approval was required. Ultimately, 508 papers were included in the analysis. The literature search strategy is detailed in Fig. 1.

### Data analysis and visualization

Developed by the research team led by Nees Jan van Eck, VOSviewer represents a widely utilized tool in the field of visual analytics. In this study, we employed VOSviewer version 1.6.20 to conduct visual analyses of international collaborations, high-frequency keywords, and author cooperation networks. Concurrently, CiteSpace software was utilized to track the burst strength trends of the top 15 keywords. Furthermore, the bibliometric package in RStudio was applied to analyze annual publication volumes, the top ten highly cited articles, and the geographical distribution of authors’ affiliated countries. Finally, Microsoft Excel 2019 was utilized to examine annual publication trends and citation dynamics. All publication volume statistics in this study were calculated using the full counting method.

## Results

### Basic overview and annual paper output

**Fig. 2:**
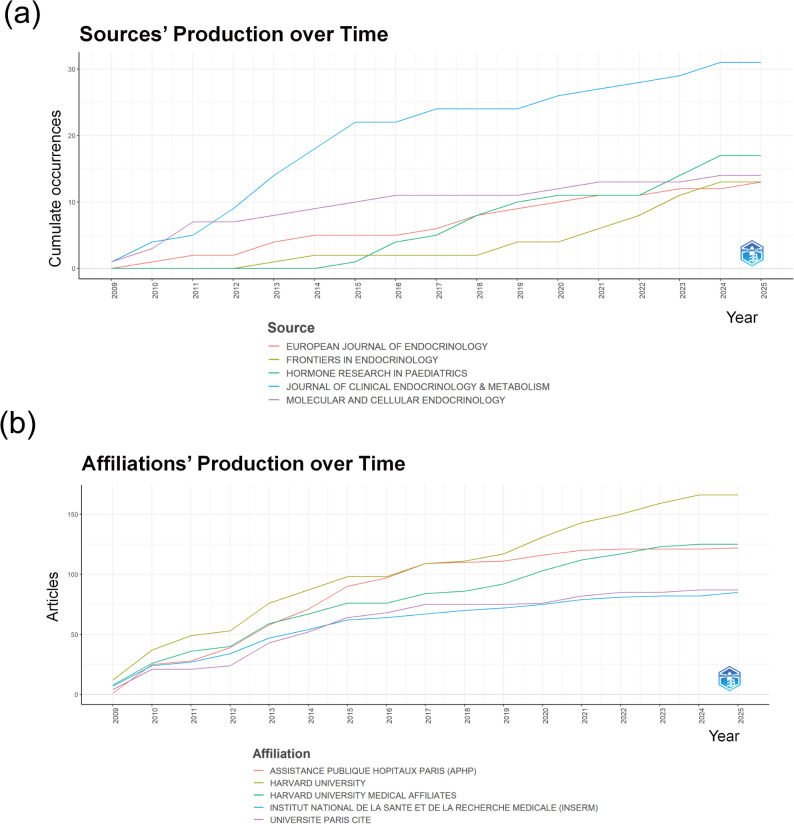
Basic overview and temporal trends in publication output. (**A**) Sources’ production over time. (**B**) Affiliations’ production over time

This study incorporated a total of 508 publications pertaining to Kallmann syndrome (KS). To investigate research trends in this field, we conducted an analysis of the annual publication output related to this topic. As illustrated in Fig. 2A and B, the number of relevant publications from various research institutions and journals has demonstrated an overall increasing trend over the years, with the *Journal of Clinical Endocrinology and Metabolism* and Harvard University ranking highest in output. These findings indicate that Kallmann syndrome (KS) remains a significant subject within the realm of rare disease research and continues to attract considerable academic attention.

### Analysis of highly cited papers

Citation count serves as a critical metric for assessing the academic impact of scholarly articles. As illustrated in Table 1, the highest citation count for a single paper reaches 575, attributed to the “Expert Consensus Document: European Consensus Statement on Congenital Hypogonadotropic Hypogonadism—Pathogenesis, Diagnosis and Treatment.” The second most-cited paper synthesizes relevant literature published prior to 2019, encompassing advancements in clinical manifestations, diagnostic evaluation, genetic diagnosis, and treatment modalities. It emphasizes that timely diagnosis and intervention can induce puberty, thereby improving bone health, metabolic function, sexual performance, and psychological well-being. Furthermore, the study highlights that pulsatile GnRH therapy can restore fertility in patients with congenital hypogonadotropic hypogonadism (CHH). The third most-cited publication investigated genes associated with hypogonadotropic disorders, identifying mutations in KISS1R, TAC3, and TACR3 as causative factors for normosmic idiopathic hypogonadotropic hypogonadism (IHH).

**Table 1:** Top 10 most-cited articles in Kallmann syndrome

Rank	Title	Citation	Author	Journal	Year	DOI
1	Expert consensus document: European Consensus Statement on congenital hypogonadotropic hypogonadism–pathogenesis, diagnosis and treatment	575	Ulrich Boehm	Nat Rev Endocrinol	2015	10.1038/nrendo.2015.112
2	Clinical Management of Congenital Hypogonadotropic Hypogonadism	256	Jacques Young	Endocr Rev	2019	10.1210/er.2018-00116
3	The genetic and molecular basis of idiopathic hypogonadotropic hypogonadism	229	Suzy D C Bianco	Nat Rev Endocrinol	2009	10.1038/nrendo.2009.177
4	The hypothalamus-pituitary-gonad axis: Tales of mice and men	224	Athina Kaprara	Metabolism	2018	10.1016/j.metabol.2017.11.018
5	Recommendations on the diagnosis, treatment and monitoring of hypogonadism in men	223	Bruno Lunenfeld	Aging Male	2015	10.3109/13685538.2015.1004049
6	Mutations in FGF17, IL17RD, DUSP6, SPRY4, and FLRT3 are identified in individuals with congenital hypogonadotropic hypogonadism	192	Hichem Miraoui	Am J Hum Genet	2013	10.1016/j.ajhg.2013.04.008
7	Kallmann syndrome	169	Catherine Dodé	Eur J Hum Genet	2008	10.1038/ejhg.2008.206
8	Neural crest and ectodermal cells intermix in the nasal placode to give rise to GnRH-1 neurons, sensory neurons, and olfactory ensheathing cells	158	Paolo Emanuele Forni	J Neurosci	2011	10.1523/JNEUROSCI.6087-10.2011
9	A genetic basis for functional hypothalamic amenorrhea	157	Lisa M Caronia	N Engl J Med	2011	10.1056/NEJMoa0911064
10	Loss-of-function mutations in SOX10 cause Kallmann syndrome with deafness	151	Veronique Pingault	Am J Hum Genet	2013	10.1016/j.ajhg.2013.03.024

### Analysis of high-output and high-cited authors

**Fig. 3:**
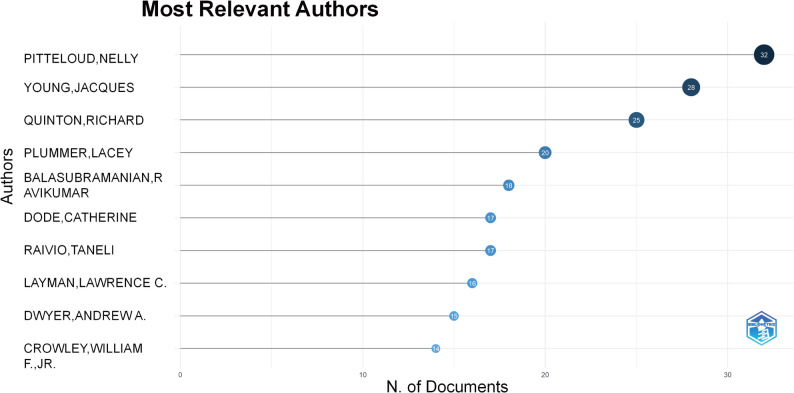
The top 10 most prolific authors in KS research

**Fig. 4:**
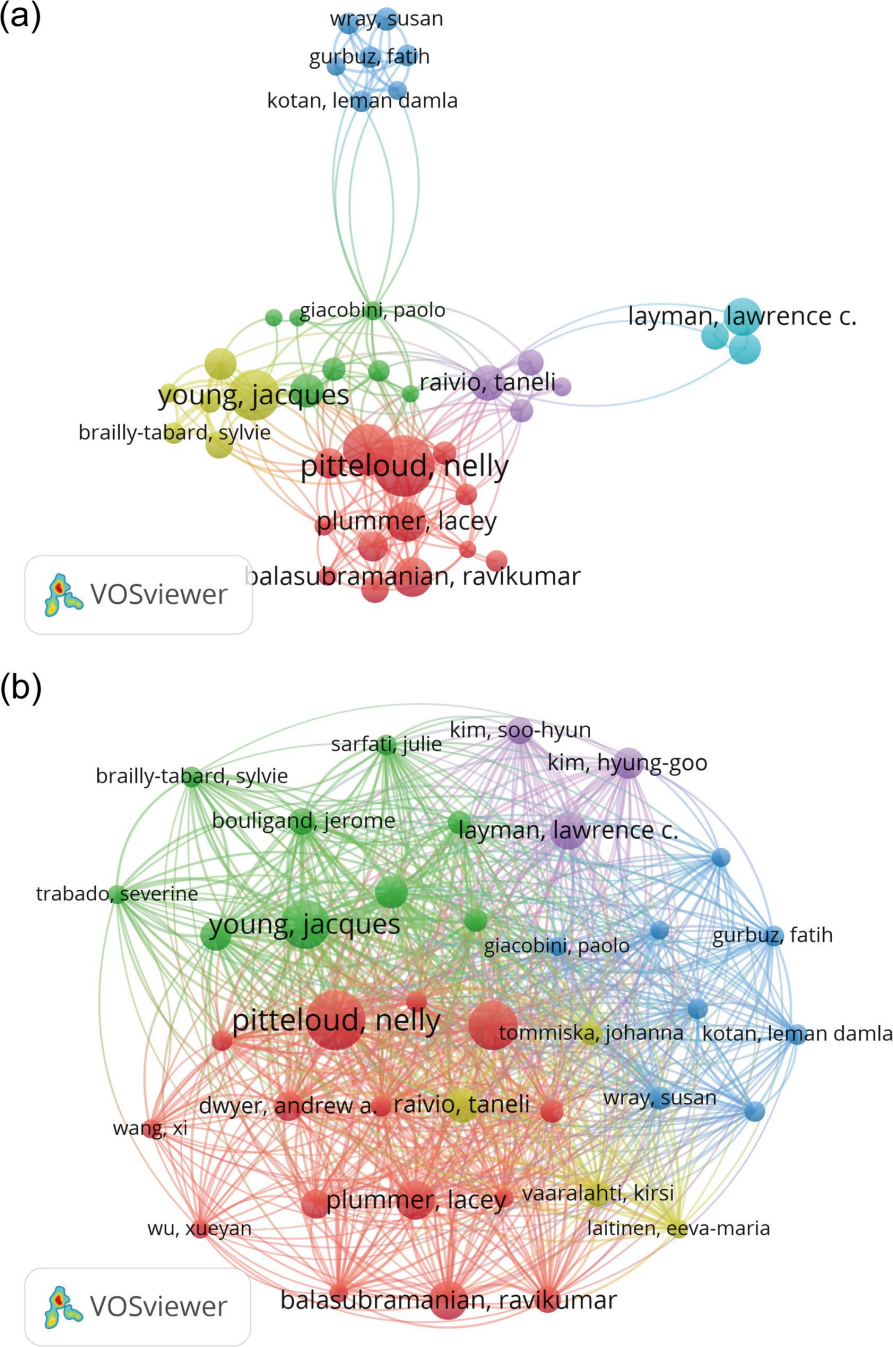
Author co-authorship and citation analysis. (**A**) Author co-authorship network generated by VOSviewer. (**B**) Author citation network generated by VOSviewer

Figure 3 illustrates the distribution of the top 10 most prolific authors in the field of KS research globally. Nelly Pitteloud from Switzerland leads with 32 publications, followed by Jacques Young from Paris Public Hospitals and Richard Quinton from Imperial College London. This indicates that Europe has developed the most concentrated academic network in KS research. As shown in (Fig. 4A), we employed VOSviewer to analyze co-authorship patterns among cited authors, presents the author co-authorship network generated by VOSviewer. To ensure analytical reliability, only authors with at least 6 publications were included, yielding 40 authors grouped into 6 distinct clusters (200 links, total link strength = 680). Node size reflects an author’s publication volume, while line thickness indicates the intensity of collaborative ties. The red cluster, anchored by core authors like Pitteloud, Nelly, represents the largest and most interconnected research team, whereas the yellow cluster (led by Young, Jacques) forms a secondary influential hub. Smaller clusters (blue, purple, green) represent specialized regional subgroups, revealing a dispersed yet collaborative academic landscap. Revealing that Professor Nelly Pitteloud maintains close collaborations with multiple scholars, including Plummer Lacey and Balasubramanian. Concurrently, Jacques Young frequently collaborates with Luigi Maione and Jérôme Bouligand. Figure 4B illustrates the author citation network constructed using VOSviewer. To ensure the reliability of the analysis, only authors with at least 7 publications were included, resulting in 39 authors distributed across 5 distinct clusters, with 688 links and a total link strength of 8405. Node size represents the citation impact of authors, while line thickness reflects the strength of citation connections between authors.The red cluster, represented by core authors including Pitteloud, Nelly and Balasubramanian, Ravikumar, exhibits the strongest citation associations and constitutes the most influential research group in the field, highlighting their central academic status. The yellow cluster led by Young, Jacques, together with smaller clusters in green, blue, and purple, corresponds to secondary influential scholars and specialized research subgroups. Collectively, the citation network displays a well-structured, distinct core-periphery architecture.The results indicate that Nelly Pitteloud and Richard Quinton occupy central positions in the network, reflecting their high citation frequency, prominent academic influence, and outstanding scholarly achievements in this field.

### Analysis of high-output countries

**Fig. 5:**
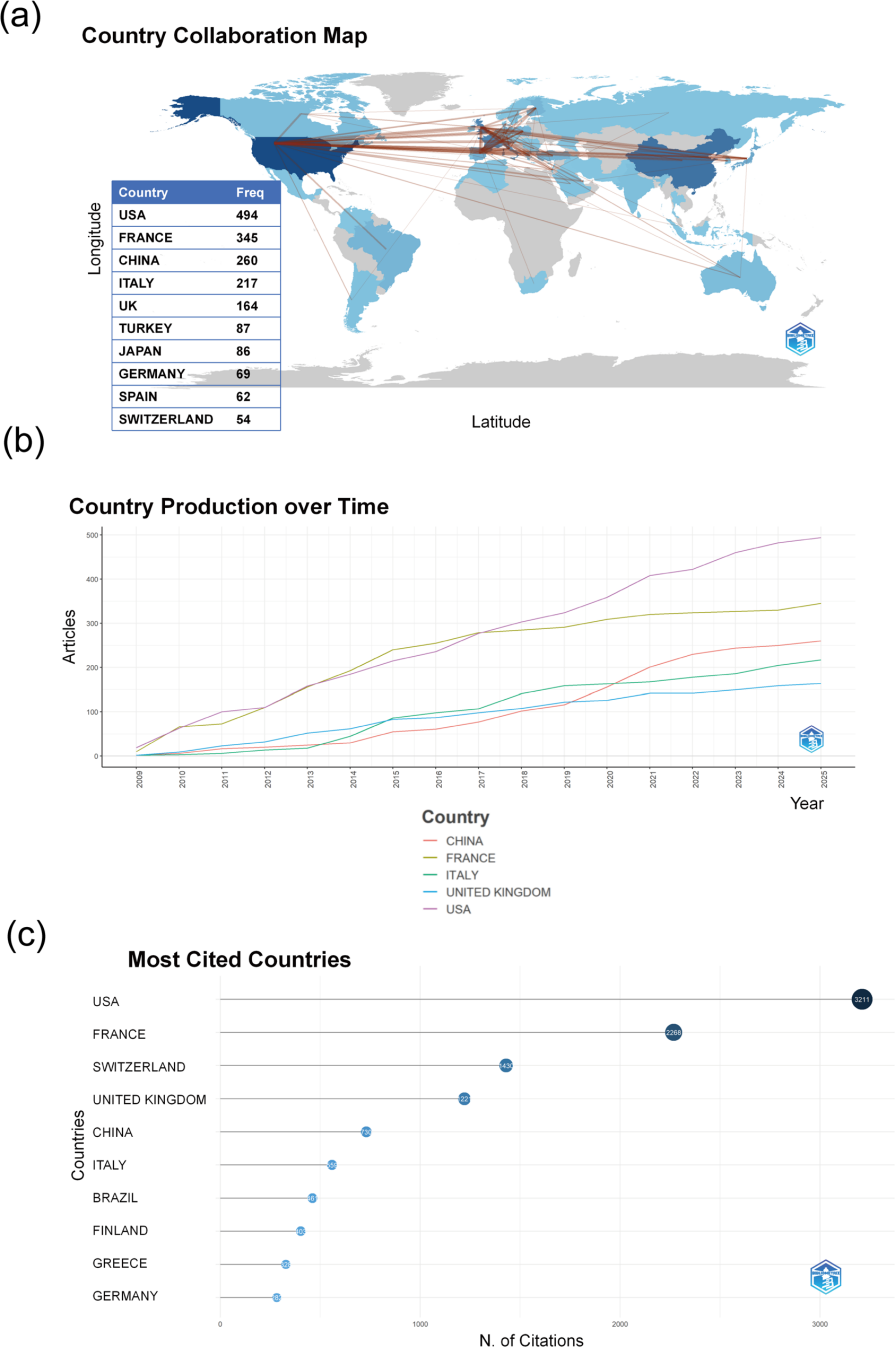
Country collaboration and publication output analysis using Biblioshiny and VOSviewer. (**A**) Country collaboration map. (**B**) Temporal trends in national publication output. (**C**) The top 10 most highly cited countries

Analysis of high-productivity countries reveals, to some extent, that the volume of publications by researchers reflects a nation’s achievements in prolific research domains. (Fig. 5A) illustrates the geographical distribution of publications and collaborative relationships among countries. Scholars from 20 nations participated in this research. As shown in (Fig. 5B), researchers from the United States contributed the highest number of publications (*N* = 494), followed by France (*N* = 345) and China (*N* = 260) (Fig. 5C). Notably, citation impact was relatively lower despite its high publication volume.

### Institutional analysis

**Fig. 6:**
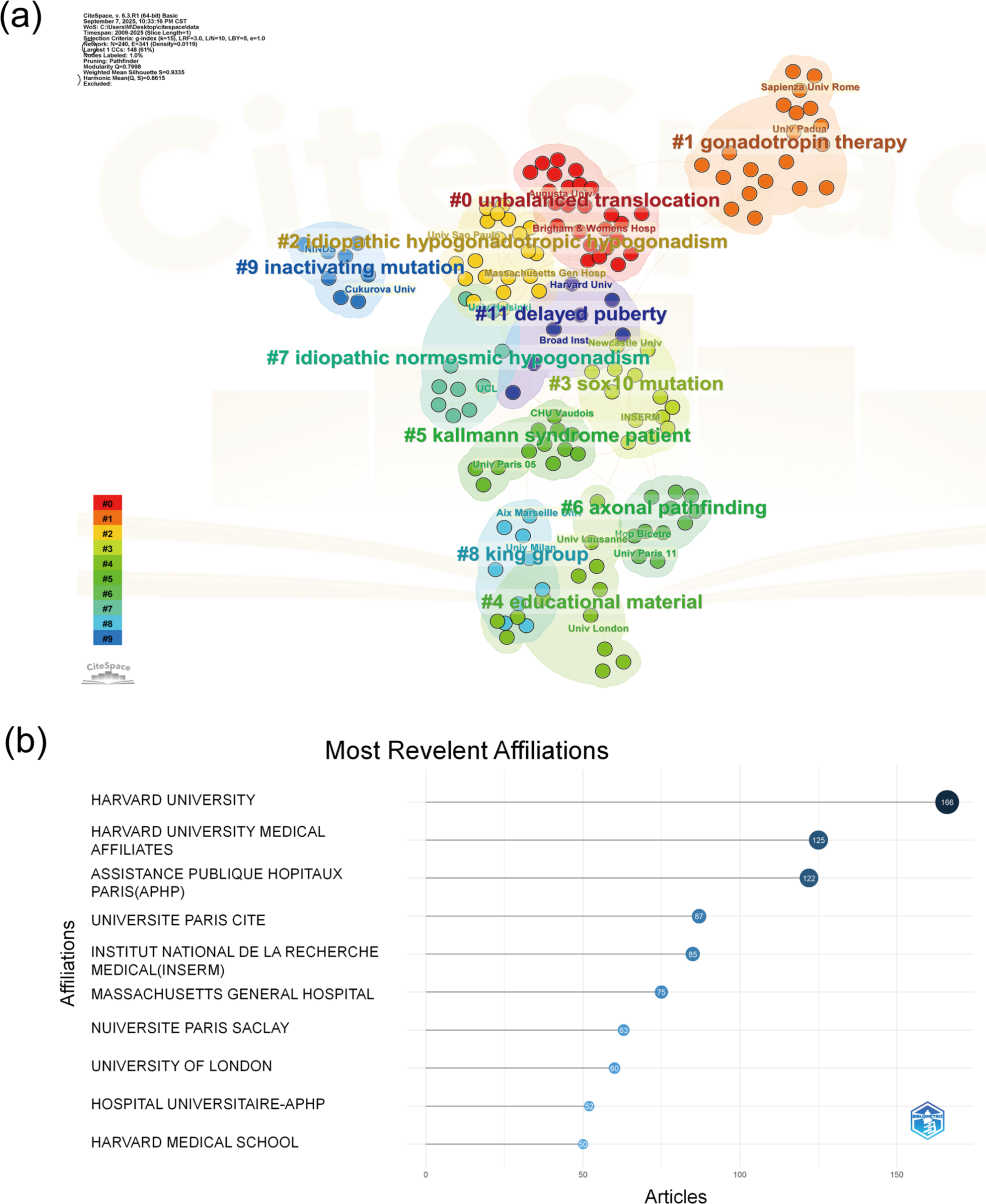
Institutional collaboration and publication output analysis using CiteSpace and Biblioshiny. (**A**) Institutional clustering network generated by CiteSpace. (**B**) The top 10 most highly cited affiliations

Figure 6A illustrates the institutional clustering network generated by CiteSpace (v.6.3.R1) for the period 2009–2025, with a slice length of 1 year. The analysis employed g-index selection criteria (k = 15), with LRF = 3.0, L/*N* = 10, LBY = 5, and e = 1.0, yielding a network of 240 nodes (institutions) and 341 edges (density = 0.0119). The modularity Q = 0.7998 and weighted mean Silhouette S = 0.9335 confirm robust clustering and clear institutional groupings. Node size reflects an institution’s publication output or citation impact, while color-coded clusters represent distinct regional and thematic research groups. Key clusters include: #0 unbalanced translocation (red): Centered on institutions like Broad Inst MIT & Harvard, representing core genomic research hubs. #5 kallmann syndrome (green): Led by institutions such as Univ Paris Sud, forming the primary clinical and basic science cluster for Kallmann syndrome. #1 gonadotropin (orange): Dominated by Seoul Natl Univ and Boston Childrens Hosp, highlighting endocrine and reproductive research centers. Smaller clusters (e.g., #9 inactivating mutations, #8 king group) represent specialized regional or thematic research subgroups. Figure 6B demonstrates that Harvard University’s publication output (*N* = 166) significantly surpasses that of both its affiliated institutions and AP-HP, indicating its comparatively stronger influence within this research domain.

### Keyword analysis

**Fig. 7:**
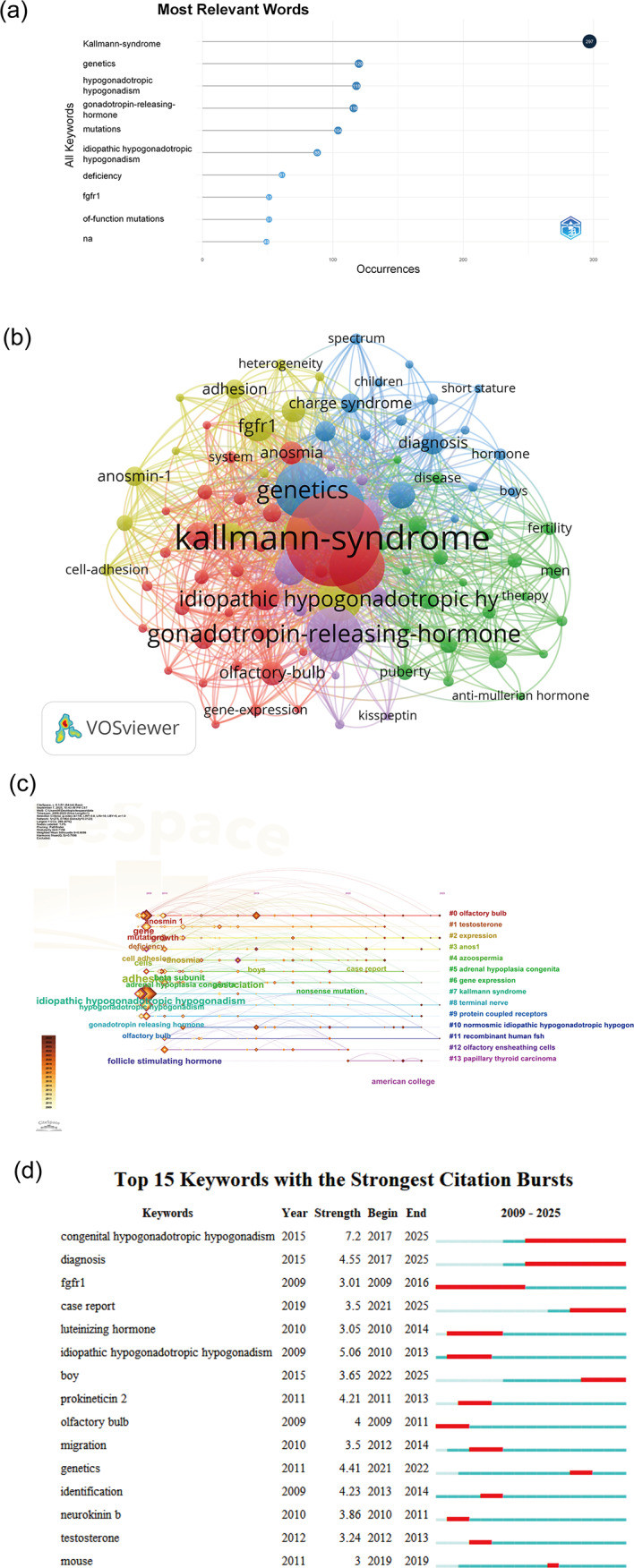
Keyword analysis. (**A**) The top 10 most frequently occurring keywords. (**B**) Keyword co-occurrence network generated by VOSviewer. (**C**) Keyword timeline visualization generated by CiteSpace. (**D**) Top 15 keywords with the strongest citation bursts

Keywords occupy a central position in any academic article. Through keyword analysis, research hotspots within the field can be identified. As illustrated in Fig. 7A, “Kallmann syndrome” and “hypogonadotropic hypogonadism” rank at the top among keywords in the domain. These are followed by “gonadotropin-releasing hormone” (*n* = 116), “mutations” (*n* = 104), “idiopathic hypogonadotropic hypogonadism” (*n* = 88), and “gene” (*n* = 75). Figure 7B visualizes the top 10 keywords using VOSviewer and presents the constructed keyword co-occurrence network. Keywords with fewer than 8 occurrences were excluded, resulting in 95 terms divided into 5 clusters (1938 links, total link strength = 6397). Node size reflects the occurrence frequency of keywords, with Kallmann syndrome as the largest central node, followed by core terms such as idiopathic hypogonadotropic hypogonadism, mutations, and genetics. Line thickness indicates the strength of keyword co-occurrence, and color-coded clusters correspond to distinct research thematic dimensions: molecular genetics, hormonal pathophysiology, and clinical manifestations.Furthermore, this study performed a visual analysis of the temporal evolution of keywords using CiteSpace (Fig. 7C). The timeline map of keywords was generated using CiteSpace (v.6.3.R1) for the period 2009–2025, with a time slice of 1 year. The g-index (k = 15) was applied as the literature screening criterion, with parameters set to LRF = 3.0, L/*N* = 10, LBY = 5, and e = 1.0. The final network comprised 275 nodes and 464 edges, with a network density of 0.0123. The modularity Q value (0.7158) and weighted mean silhouette S value (0.9056) verified the reliability of clustering results and clear delineation of research themes.The timeline map identified several distinct research front clusters, including Kallmann syndrome phenotype, anosmin-1 interaction, gonadotropin-releasing hormone, and clinical management. In the map, nodes represent keywords, and node size corresponds to citation burst strength; colored lines connect research themes across periods, clearly revealing the evolutionary trend of research hotspots in this field from molecular genetics (e.g., prokineticin receptor, heterozygous deletion) toward clinical applications (e.g., clinical management, genetic counseling). Another significant indicator reflecting the evolution of research frontiers and hotspots is the burst strength of keywords (Fig. 7D). Among the top 15 keywords with the highest citation burst strength, “congenital gonadotropin-releasing hormone deficiency” exhibited the strongest burst (7.2), followed by “idiopathic gonadotropin-releasing hormone deficiency” (5.06). It is noteworthy that the burst of keywords such as “diagnosis,” “case report,” “boys,” and “congenital gonadotropin-releasing hormone deficiency” continues through 2025, indicating that these topics remain highly active.

### Citation analysis

**Fig. 8:**
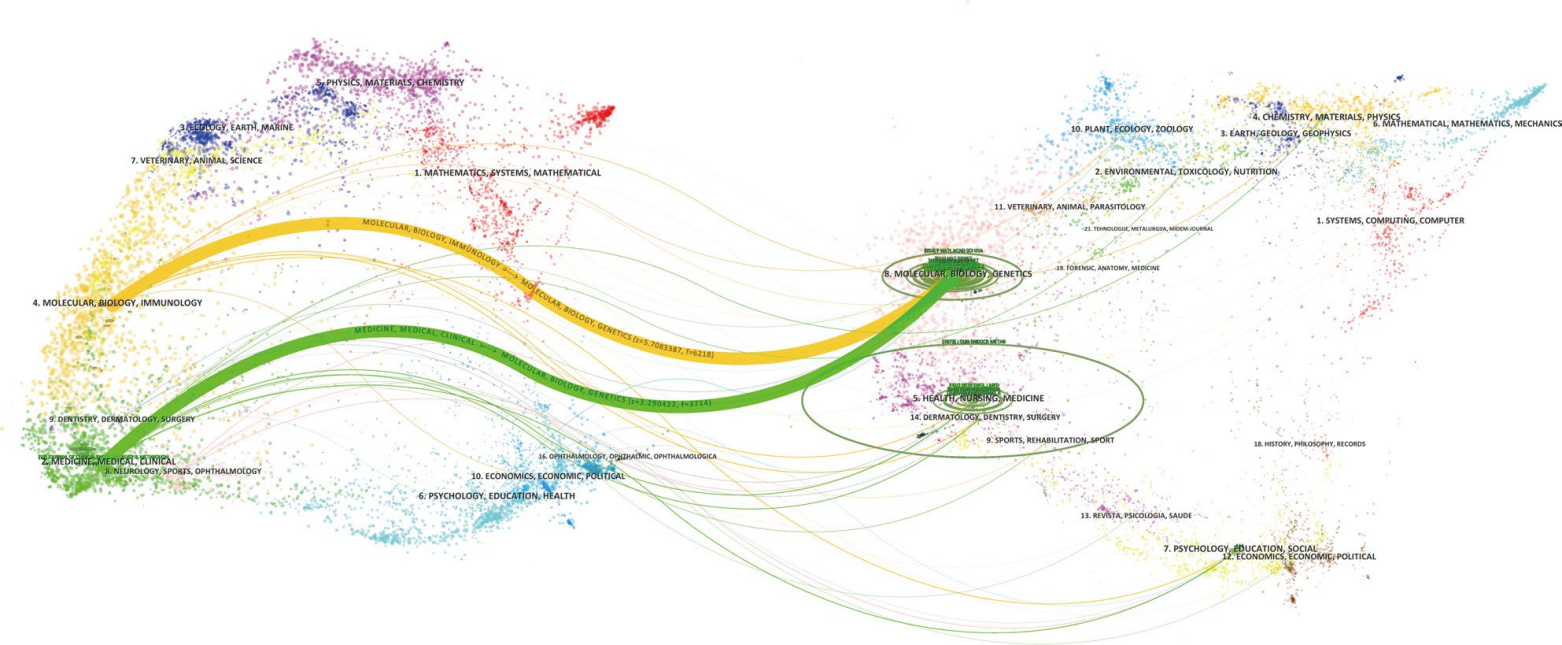
Dual-map overlay of journal disciplines in KS research

Based on citation analysis, this study unraveled the thematic distribution of journals via the dual-map overlay illustrated in Fig. 8. Generated by CiteSpace, this disciplinary dual-map overlay vividly characterizes the disciplinary citation landscape and knowledge flow patterns within the research field. In the overlay, nodes represent individual journals, which are colored in accordance with their subject categories, whereas curved connecting lines signify the citation linkages across different disciplines.The map delineates two primary disciplinary zones: the **left zone (citing disciplines)** features dense clusters encompassing Medicine, Medical, Clinical (green), Molecular, Biology, Immunology (yellow), and Mathematics, Systems, Mathematical (red), as well as applied disciplines such as Veterinary, Animal Science, and Dentistry, Dermatology. The **right zone (cited disciplines)** is dominated by core recipient clusters including Molecular, Biology, Genetics (green) and Health, Nursing, Medicine (purple), accompanied by secondary clusters in Chemistry, Materials Science and Earth, Geology.Notably, the thick colored lines highlight the dominant knowledge transfer pathways: a striking green pathway runs from Medicine, Medical, Clinical to Molecular, Biology, Genetics, and a yellow pathway extends from Molecular, Biology, Immunology to the same core cited cluster. These pathways indicate intensive interdisciplinary interactions and knowledge integration between clinical medicine and molecular life sciences.

## Discussion

Bibliometric analysis provides a rigorous, data-driven approach to synthesizing large scholarly corpora, clarifying temporal trends, collaborative patterns, and knowledge frontiers in specialized domains such as rare genetic disorders [13, 15]. To our knowledge, this is the first comprehensive bibliometric analysis of KS, which systematically analyzes 508 publications indexed in the Web of Science Core Collection (WOSCC) from 2009 to 2025. We analyzed the research landscape of KS from multiple perspectives, including country distribution, institutional collaboration, and research hotspots, using VOSviewer, CiteSpace, and R software for visualization and result interpretation.

### Global publication landscape

Publication volume serves as a key measure of research activity in rare diseases, for which large-scale clinical investigations are often constrained by low disease prevalence [16, 17]. Our findings chronicle a robust escalation in KS outputs, manifesting as consistent double-digit annual growth rates and culminating in 508 vetted studies (from an initial pool of 850, post-exclusion of non-KS hypogonadisms like Klinefelter or CHARGE syndromes). This trajectory resonates with broader rare disease publication surges, underscoring amplified academic traction amid global initiatives like the International Rare Diseases Research Consortium (IRDiRC). Geospatially, the United States spearheads contributions, trailed by Switzerland and France, mirroring affiliation patterns where U.S. authors helm 40% of KS literature since 2009. This hegemony likely stems from robust funding ecosystems (e.g., NIH Rare Diseases Clinical Research Network) and expertise hubs. Institutional centrality analysis spotlights Harvard University as paramount, boasting the highest output and network influence, emblematic of its preeminence in integrative endocrinology-genetics paradigms. Authorial networks evince dense transatlantic and Euro-Asian linkages, with luminaries like Nelly Pitteloud (Switzerland) and Jacques Young (France) accruing pivotal citations for seminal works on genetic etiology and consensus frameworks [20]. In the co-authorship network, the red cluster led by Pitteloud, Nelly is the dominant high-yield core, and the yellow cluster headed by Young, Jacques is another pivotal hub, representing parallel core teams. Six discrete clusters form a modular collaboration framework with favorable academic diversity, and moderate inter-cluster links suggest boosted interdisciplinary research via closer cross-team cooperation. The citation network further defines the field’s intellectual core: the cluster led by Pitteloud, Nelly serves as the foundational reference, and secondary clusters cover targeted research directions with close cross-branch exchanges.The institutional collaboration network reveals the global research cooperation pattern of Kallmann syndrome. High modularity and silhouette coefficients verify distinct clustering, reflecting geographic agglomeration and specialized research division. The green cluster (#5 kallmann syndrome) centered at European institutions (e.g., Université Paris-Sud) dominates the field, leading fundamental and clinical research. The red cluster (#0 unbalanced translocation) and orange cluster (#1 gonadotropin) serve as complementary hubs for genomic and endocrinological studies, highlighting interdisciplinary collaboration. Smaller clusters highlight regional strengths, forming a globally interconnected research ecosystem. This structure shows the field relies on core institutional hubs with diverse regional contributions, indicating that intensified international cooperation can accelerate clinical translation. Such interconnections—spanning the Americas, Europe, and Asia (notably China)—foster resource pooling essential for surmounting KS’s diagnostic odyssey, often protracted by phenotypic variability and limited cohort sizes. Journal distributions affirm endocrine-centric foci, with the Journal of Clinical Endocrinology and Metabolism commanding paramount volume, while interdisciplinary venues like Orphanet Journal of Rare Diseases signal emergent cross-pollination with neurology and reproductive medicine. Collectively, these patterns advocate for intensified multilateral collaborations to democratize access to high-caliber insights, thereby mitigating disparities in rare disease management across resource-constrained settings. The keyword co-occurrence network reveals the intellectual structure of Kallmann syndrome research. The centrality of “Kallmann syndrome” confirms its status as the core research subject, while adjacent high-frequency terms such as “mutations” and “genetics” highlight the field’s strong emphasis on molecular etiology. The prominence of “idiopathic hypogonadotropic hypogonadism” and “gonadotropin-releasing hormone” underscores the central role of hormonal pathophysiology, whereas terms including “diagnosis” and “puberty” reflect key directions in clinical and translational research.Five distinct clusters demonstrate a modular research landscape, achieving the organic integration of basic research (genetics, molecular pathways) and clinical practice.Timeline analysis unravels the 16-year dynamic evolution of Kallmann syndrome research. Early clusters (2009–2013) focused on molecular etiology, centered on key genes including anosmin-1 and prokineticin receptors, marking groundbreaking discoveries in genetic pathogenesis. Mid-term clusters (2014–2020) shifted focus to hormonal signaling pathways (e.g., gonadotropin-releasing hormone pathway) and clinical translation. Recent research (2021–2025) has concentrated on clinical management and genetic counseling. Elevated modularity and silhouette coefficients confirm a well-defined thematic structure, demonstrating a progressive transition from basic science to patient-centered research.

### Background in clinical and genetic research

Kallmann syndrome (KS) is a monogenic inherited disorder defined by the co-occurrence of congenital hypogonadotropic hypogonadism (CHH) and hyposmia or anosmia, which arises from impaired differentiation and migration of embryonic gonadotropin-releasing hormone (GnRH) neurons from the developing olfactory placode to the hypothalamus during embryogenesis [18]. Genetically, KS is characterized by extreme heterogeneity, with both sporadic and familial inheritance patterns reported; approximately 60% of affected cases are sporadic. Affected individuals display substantial variability in both genotypic and phenotypic profiles, rendering genetic testing a cornerstone for definitive diagnosis and personalized clinical decision-making. To date, rare sequence variants in known pathogenic genes have been identified in approximately 40% of KS patients [19], while the molecular etiology remains unresolved in the remaining majority of cases. Clinically, the mainstay of KS management centers on hormone replacement therapy and fertility induction; with timely diagnosis and early intervention, including pulsatile GnRH infusion or gonadotropin treatment, reproductive potential can be successfully restored in most affected individuals [20]. In recent years, successive updates to clinical practice guidelines from the Endocrine Society have driven growing emphasis on standardized early screening, formal diagnostic criteria, long-term systematic follow-up, and health-related quality of life for KS patients. This evolving clinical priority aligns directly with the emerging frontier of clinical management identified in our bibliometric analysis, and collectively, these well-established advances in the genetic and clinical landscape of KS provide a robust biological and clinical rationale for the shifting research priorities we observed.

### Hotspots and frontiers

Keyword co-occurrence and burst analytics demystify thematic nuclei, unveiling hotspots that encapsulate KS’s core pathophysiological and clinical contours [21, 22].Kallmann syndrome (KS) has a strong genetic basis and is caused by hereditary factors, which can be attributed to mutations in a variety of distinct genes. Although the majority of KS cases are sporadic, many cases are clearly familial, and more than 30 mutated genes in KS patients have been identified to date [3]. These genes either act alone (autosomal dominant [AD], autosomal recessive [AR], and X-linked inheritance patterns) or in combination (digenic or oligogenic modes) [23]. Clinical genetic testing can identify the particular pathogenic gene responsible for an individual patient’s disease [4]. Recent advances have been achieved in next-generation sequencing, which enables massively parallel sequencing of multiple samples [24], as well as in bioinformatics analyses targeting the expression of KS-related pathogenic genes in olfactory cells or gonadotropin-releasing hormone (GnRH) neurons. These advances will facilitate the discovery of novel factors involved in the physiological activities of GnRH neurons and the pathogenesis and progression of KS. In addition, prospective longitudinal cohort studies are warranted to establish standardized early screening strategies for high-risk populations, optimize individualized treatment regimens, and evaluate the long-term prognosis, fertility outcomes, and psychological status of KS patients, so as to promote the translation of basic research findings into precise clinical management. These prospective directions are expected to fill the current gaps in KS research and foster high-quality development in this field.

## Conclusion

This study presents a comprehensive bibliometric analysis to systematically delineate the global research landscape of Kallmann syndrome (KS) over the past 15 years. By integrating visualization and statistical tools including VOSviewer, CiteSpace and R software, we performed a systematic analysis of 508 eligible publications retrieved from the Web of Science Core Collection spanning 2009 to 2025. Our analysis verified a steady upward trend in research output pertaining to KS, identified the core publishing countries, high-profile research institutions, influential authors and top-tier journals in this field, and further elucidated the evolutionary trajectories of research themes.The results revealed a consistent year-on-year rise in global KS-related publications. The United States, Switzerland and France were the major contributing countries, while Harvard University ranked as the leading research institution. The bulk of relevant studies were published in authoritative journals specializing in endocrinology, and well-established clinical practice guidelines have laid a solid foundation for subsequent investigations in this field. Our findings further clarify the global research layout of KS, which is predominantly dominated by European and American countries, and underscore cross-regional cooperation as a pivotal impetus to propel field advancement. Collectively, these results offer new insights for related research and provide useful guidance for investigators in future work.

## Limitation

This study has some limitations. First, our analysis was restricted to publications indexed in the Web of Science Core Collection (WOSCC), which introduces database-specific indexing bias and limits the scope of retrieved literature, as relevant studies indexed in alternative databases (including PubMed, Embase, and Scopus) or published in non-SCI journals were excluded. Second, we only included English-language articles, resulting in an English-centric bias that may underrepresent non-Western and multilingual research. Third, the adopted search strategy carries potential risks to search sensitivity and specificity, meaning some eligible publications may have been missed while a small number of marginally irrelevant articles were retained. Fourth, bibliometric outcomes are highly reliant on the accuracy and consistency of database metadata, including discrepancies in author names, institutional affiliations, and keyword tagging, all of which can compromise analytical precision. Fifth, citation-based metrics are inherently affected by citation lag, which favors older publications and risks underestimating the impact of recently published work in this field. Sixth, owing to overlapping terminology and blurred research boundaries, it remains difficult to strictly delineate KS-specific literature from the broader CHH-related studies. Seventh, varying publication counting approaches (such as whole counting versus fractional counting) can alter country and institutional ranking outcomes, adding further constraints to result interpretation. Additionally, co-citation and co-occurrence analyses are prone to citation-related biases including publication bias, self-citation, authorship bias, and journal impact factor bias, which may undermine result objectivity; limiting data extraction to first authors and inconsistencies in keyword phrasing further reduce the stability of network analysis. Despite these limitations, the core research trends identified in this study are robust and offer meaningful guidance for future investigations. 

## Electronic Supplementary Material

Below is the link to the electronic supplementary material.

Supplementary Material 1

## Data Availability

All data for this study are available from the Web of Science Core Collection database.
